# In Vivo Analysis of miR-34a Regulated Glucose Metabolism Related Genes in *Megalobrama amblycephala*

**DOI:** 10.3390/ijms19082417

**Published:** 2018-08-16

**Authors:** Ling-Hong Miao, Yan Lin, Xin Huang, Wen-Jing Pan, Qun-Lan Zhou, Bo Liu, Ming-Chun Ren, Xian-Ping Ge, Liang-Kun Pan

**Affiliations:** 1 Key Laboratory of Freshwater Fisheries and Germplasm Resources Utilization, Ministry of Agriculture, Freshwater Fisheries Research Center, Chinese Academy of Fishery Sciences, Wuxi 214081, China; miaolh@ffrc.cn (L.-H.M.); liny@ffrc.cn (Y.L.); zhouql@ffrc.cn (Q.-L.Z.); liub@ffrc.cn (B.L.); renmc@ffrc.cn (M.-C.R.); panlk@ffrc.cn (L.-K.P.); 2 Wuxi Fisheries College, Nanjing Agricultural University, Wuxi 214081, China; 17798746965@163.com (X.H.); pan787856_wj@163.com (W.-J.P.)

**Keywords:** *Megalobrama amblycephala*, miR-34a, inhibition, glucose metabolism, RNA-seq

## Abstract

The *Megalobrama amblycephala* (*M. amblycephala*) is one of the most important economic freshwater fish in China. The molecular mechanism under the glucose intolerance responses which affects the growth performance and feed utilization is still confused. miR-34a was reported as a key regulator in the glucose metabolism, but how did the miR-34a exert its function in the metabolism of glucose/insulin in *M. amblycephala* was still unclear. In this study, we intraperitoneally injected the miR-34a inhibitor (80 nmol/100 g body weight) into *M. amblycephala* (fed with high starch diet, 45% starch) for 12 h, and then analyzed the gene expression profiling in livers by RNA-seq. The results showed that miR-34a expression in *M. amblycephala* livers was inhibited by injection of miR-34a inhibitor, and a total of 2212 differentially expressed genes (DEGs) were dysregulated (including 1183 up- and 1029 downregulated DEGs). Function enrichment analysis of DEGs showed that most of them were enriched in the peroxisome proliferator-activated receptor (*PPAR*), insulin, AMP-activated protein kinase (*AMPK*) and janus kinase/signal transducers and activators of transcription (JAK/STAT) signaling pathways, which were all associated with the glucose/lipid metabolic and biosynthetic processes. In addition, we examined and verified the differential expression levels of some genes involved in AMPK signaling pathway by qRT-PCR. These results demonstrated that the inhibition of miR-34a might regulate glucose metabolism in *M. amblycephala* through downstream target genes.

## 1. Introduction

Carbohydrates are the main source of energy in most animal diets [1], and its properties such as digestion and absorption rate, viscosity, structural features, water-binding capacity and fermentation ability in the gastrointestinal tract are of critical importance in the effect of nutrition [2]. It has been reported that the utilization of dietary carbohydrates and their effects on growth and nutrient deposition are very important [3], and the dietary carbohydrate inclusion in several fish species appears to produce positive effects on growth and digestibility [4,5,6].

Blunt snout bream (*Megalobrama amblycephala*, *M. amblycephala*) is one of the most important economic freshwater fish in China [7]. According to the latest statistics from Food and Agriculture Organization (FAO), the total output of *M. amblycephala* reached nearly 850 thousand tons in 2016, and the market demand is increasing due to its high economic value. *M. amblycephala* is typical herbivorous feeding habit [8], and its digestive function and disease resistance ability are related to high-fat and -glucose diets [9]. Researches have shown that higher carbohydrate dietary formula (>34%) might result into glucose intolerance responses in *M. amblycephala* [10,11,12]. But the underlying molecular mechanism is still unclear. Our previous study provided a miRNA profiling in response to high starch treatment in *M. amblycephala*, and showed that miRNAs might play crucial roles in glucose metabolism [9]. In that study, 124 differentially expressed miRNAs (DEMs) were identified in liver tissue between fishes fed with normal and high starch diets and a noteworthy upregulated miRNA, miR-34a, was found in the DEMs list [9]. There might be association between miR-34a upregulation and glucose metabolism.

miR-34a, a highly conserved, endogenous, small non-coding RNA, has emerged as a key biomarker in cellular senescence and tumor suppression through its interaction with *SIRT1* (Sirtuin 1) and *p53* (Tumor protein p53) [13,14]. miR-34a has additionally been implicated in diabetes as a target to prevent pancreatic β-cell death [15,16]. Several studies had reported that miR-34a was dysregulated in metabolic tissues in rodent models and human patients of obesity, type 2 diabetes (T2D) and Non-Alcoholic Fatty Liver Disease (NAFLD) [17,18,19,20,21]. MiR-34a was highly elevated in liver in NAFLD and T2D patients compared to healthy controls [20,21]. Previous studies suggested that miR-34a regulated fat metabolism and insulin secretion by targeting *SIRT1* and acyl-CoA synthetase long-chain family member 1 (*ACSL1*) [15,22,23]. *SIRT1* is a key metabolic sensor and regulator in cells and directly deacetylates and modulates some important metabolic regulators, including *PGC-1α* (peroxisome proliferator-activated receptor γ coactivator 1-α), peroxisome proliferator-activated receptors (*PPARs*), *p53*, and forkhead box protein O1 (*FOXO1*) which change the expression of transcriptional programs that modulate cholesterol, lipid and energy homeostasis [24]. Moreover, researchers demonstrated that miR-34a was involved in insulin hyposecretion, insulin resistance, and cell injury in a mammalian T2D model [25]. Therefore, we hypothesized that miR-34a might play an important role in promoting glucose intolerance in human and animals.

Researches on the dysregulation of miR-34a in glucose metabolism have been widely reported in rodents and human [26,27], but little is known in the teleosts yet, especially in *M. amblycephala*. Hence, we used next-generation sequencing technologies to identify the potential regulatory networks of miR-34a in *M. amblycephala.* In this study, we conducted an in vivo experiment by injecting the inhibitor of miR-34a into *M. amblycephala*, and then constructed three poly(A)^+^ libraries from livers in consideration of the suggestion that the main target organ of miR-34a was liver tissue [28]. After sequencing and bioinformatics analysis, a number of differentially expressed genes (DEGs) including the target genes of miR-34a would be identified. These results may provide new insights into the regulatory mechanism of miR-34a in high-glucose metabolism in *M. amblycephala*.

## 2. Results

### 2.1. Transcriptome Sequencing in M. amblycephala

**Figure 1:**
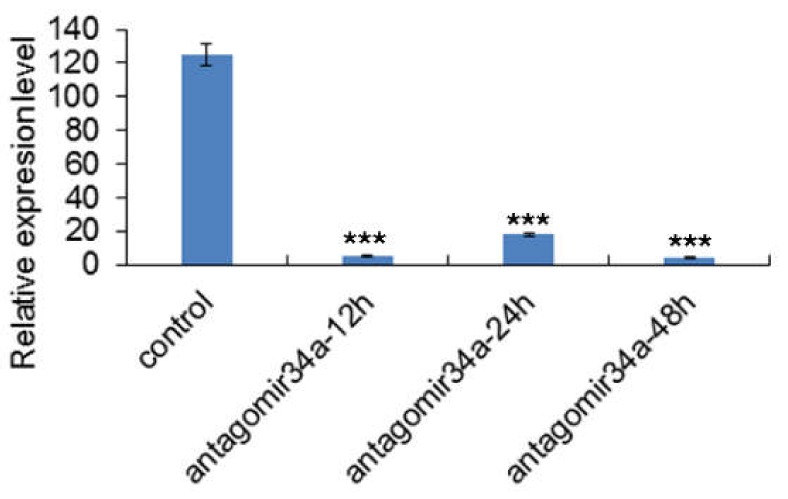
The relative expression level of miR-34a in livers of fishes treated with high starch diet. All fishes were treated with 80 nmol/100 g body weight miR-34a inhibitors (antagomiR-34a) for 12, 24, and 48 h, combined with high starch diet (45% wheat starch). Relative expression levels of miR-34a was detected using qRT-PCR. *** notes *p* < 0.001 vs. control.

Our results suggested that the injection of miR-34a inhibitor (80 nmol/100 g body weight) for 12, 24, and 48 h showed significant effect on miR-34a expression level in livers of fishes treated with high starch diet (HSD, 45% wheat starch, Figure 1, *p* < 0.001). So we performed RNA-seq analysis using the liver samples from fishes treated with antagomiR-34a for 12 h. Three poly(A)^+^ RNA-seq libraries were constructed and sequenced using paired-end (PE 2 × 150) Illumina technique. We obtained total 145.5 million pair-end (PE) reads (raw data) from three libraries (Table 1), and 143.6 million reads (clean data) were remained after the removal of ambiguous nucleotides and low-quality sequences, and all the further analyses were performed based on the clean data. RNA-seq data generated in this study had been deposited in the NCBI SRA database (SRP110655).

### 2.2. De Novo Assembly of the M. amblycephala Transcriptome

After quality control process, de novo assembly for clean short reads generated from all samples was accomplished by Trinity software with default parameters. Trinity produced 222,411 contigs, corresponding to 197,425 unique genes, with ranged length from 200 to 16,839 bp and N50 of 1041 (Table 2). Length distribution analysis displayed that most contigs were in the range of 200–1000 bp, and only 14.78% (32,879) contigs ranged from 1000 to 16,839 bp (Figure S1).

### 2.3. Functional Annotation

**Figure 2:**
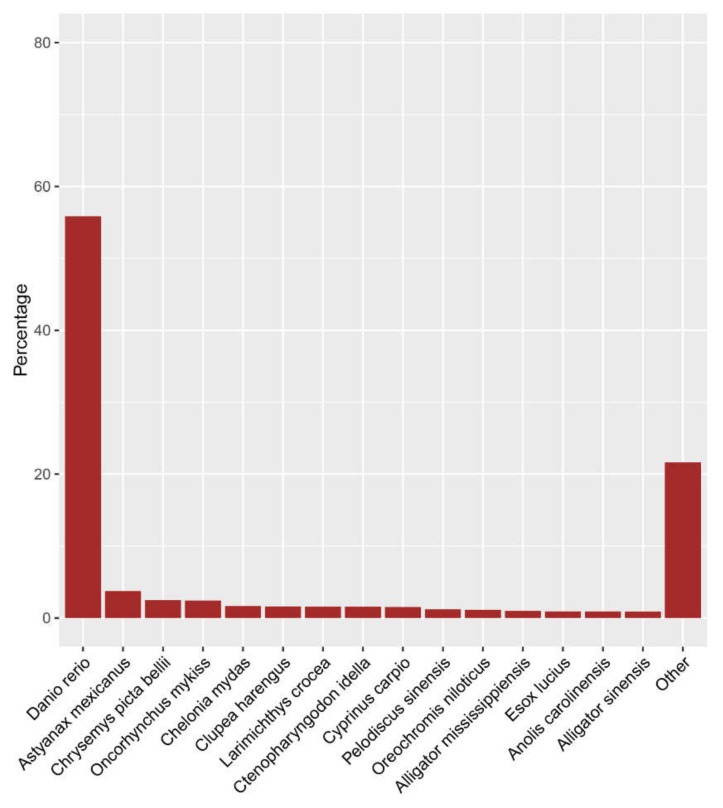
Homologous species distribution of the annotated contigs.

To annotate the *M. amblycephala* transcriptome and identify genes, we performed a functional annotation on the assembly using BLASTx alignment with a cut-off *e*-value < 1 × 10^−10^. All assembled contigs were searched against the Nr database, UniProt-SwissProt database, COG (Cluster of Orthologous Groups of protein) database, the KEGG (Kyoto Encyclopedia of Genes and Genomes), and Protein Family (Pfam) conserved domain database, respectively. A total of 83,587 cumulatively assembled contigs, corresponding to the prediction of 67,604 unique genes (Table 3), were identified. There were 82,223 assembled contigs with significant hits against Nr database and 87.33% contigs with an *e*-value of <1 × 10^−10^ (Figure S2). Most of the annotated contigs were homologous to the *Danio rerio* (55.85%) (Figure 2), followed by *Astyanax mexicanus* (3.73%) and *Chrysemys picta bellii* (2.48%). Moreover, 62.42% of the assembly contigs could not map to any known proteins. We speculated that some of these sequences were *M. amblycephala* lineage-specific genes or non-coding RNAs and assembly noises.

The KEGG analysis helps us to understand the network of molecular interactions in cells in *M. amblycephala*. Based on the comparison against the KEGG database, 71,045 contigs were annotated and enriched into 322 KEGG pathways, including phosphatidylinositol3-kinase/protein kinase B (PI3K-Akt), MAPK, and insulin signaling pathways (Table S1).

Gene ontology (GO) annotation was then performed based on the Nr annotation and 25,433 contigs (11.44%) were assigned to 52 GO terms, including 22 biological process terms, 18 cellular component terms and 12 molecular function terms. In the biological process category, cellular process (11,164 genes) was the most abundant term, followed by single-organism process term (10,652 genes) and metabolic process term (8833 genes). For the cellular component category, cell and cell part terms enriched 8274 unigenes. Within the molecular function category, binding (10,508 genes) was the most predominant term (Table S1 and Figure S3).

With the COG annotation, the 45,994 contigs were annotated to the 25 COG function clusters (Table S1 and Figure S4). Among the functional classes, the largest cluster was the general function prediction only (19.77%), followed by signal transduction mechanisms (15.77%), posttranslational modification, protein turnover, chaperones (9.7%). Only a few contigs were assigned to cell motility and nuclear structure (Table S1 and Figure S4).

### 2.4. Identification of Simple Sequence Repeat

Simple sequence repeat (SSR) markers are locus-specific, tandem, abundant and highly polymorphic in genome and have been proven to enable the development of molecular markers in genetics and breeding. To discover the SSRs markers in the contigs of *M. amblycephala* in our study, assembled contigs were used to identify potential microsatellite motifs using MIcroSAtellite (MISA) software. A total of 92,834 SSRs were identified in 61,845 contigs and 20,733 contigs (33.52%) having more than one SSR (Table 4 and Table S2). It means that there is a SSR marker per 1.6 kb in the *M. amblycephala* transcriptome. With the assuming that mononucleotide repeats may be caused by the sequencing errors and assembly mistakes, we excluded 62,185 detected mono-nucleotide repeats. Accordingly, the most abundant type of repeat motifs was dinucleotide (22.02%), followed by trinucleotide (8.10%), tetranucleotide (2.42%), pentanucleotide (0.44%) and hexanucleotide (0.04%) repeats. Future research direction is to discover reliable markers in *M. amblycephala*. The more information of the SSR was in the Table S2.

### 2.5. Open Reading Frame Identification and Prediction

**Figure 3:**
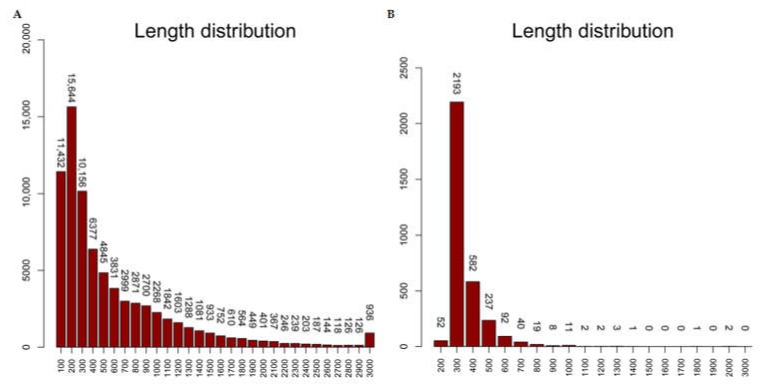
Open reading frame (ORF) length distribution of the annotated contigs (**A**) and the predicted ORF length distribution of the unannotated contigs (**B**) in *M. amblycephala*.

For the assembled contigs which had hits against Nr database, there were 75,338 annotated contigs were found containing the ORF (Open Reading Frame), with a range of 102–13,158 bp and an average of 646.79 bp (Figure 3A). The Transdecoder program was performed to detect the potential ORF regions in the remaining unannotated contigs. Results showed that there were 3245 contigs containing predicted ORF region, with an average, minimum, and maximum length of 394.29, 297 and 5737 bp, respectively (Figure 3B). There were 143,828 contigs containing no ORF regions, which suggested that they might come from non-coding genes or uncompleted assembled untranslated regions (UTR).

### 2.6. DEG Predication and Clustering Analysis

**Figure 4:**
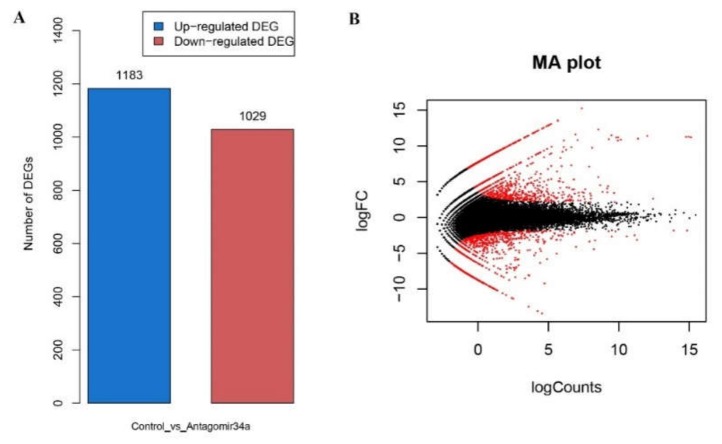
Differentially expressed genes in *M. amblycephala* in response to miR-34a inhibitor treatment. (**A**) Blue represents upregulated genes and red represents the downregulated genes in antagomiR34a-12h group vs. control group. |log2FC(Fold change)| ≥ 1 and *p*-value ≤ 0.05; (**B**) MA plot of all DEGs, the *y*-axis represents the logarithm of fold change and the *x*-axis represents the logarithm of read counts. Red color represents DEGs and black color represents non-DEGs.

With the criteria of |LogFC (Fold change)| ≥ 1 and FDR (False Discovery Rate) ≤ 0.05, we identified 2212 DEGs, including 1183 (53.55%) upregulated DEGs and 1029 (46.45%) downregulated DEGs, in antagomiR34a-12h samples comparing with the control (Figure 4). In the list of all DEGs, we found that *PIK3R1* (phosphatidylinositol 3-kinase regulatory subunit α), *PEPCK* (phosphoenolpyruvate carboxykinase), *IL6RA* (interleukin-6 receptor subunit α), *INSIG1* (insulin-induced gene 1 protein), *MHCI* (major histocompatibility complex class I), *MHCII* (major histocompatibility complex class II), *GADD45B* (Growth arrest and DNA-damage-inducible, β), *CES1* (Carboxylesterase 2), *HDAC10* (Histone deacetylase 10), *ACC1* (acetyl-CoA carboxylase 1), *CREBBP* (CREB-binding protein isoform X3), *PGC-1α* (peroxisome proliferator-activated receptor γ coactivator 1-α), and *HMGCR* (3-hydroxy-3-methylglutaryl-coenzyme A reductase) were dysregulated in the antagomiR34a-12h samples comparing with control (Table S3). All these genes were directly or indirectly responded to glucose and insulin metabolism [24]. Hierarchical clustering and heat mapping of the DEGs indicated the different expression profiles of all DEGs in livers between control and antagomiR34a-12h group (see Figure S5).

**Figure 5:**
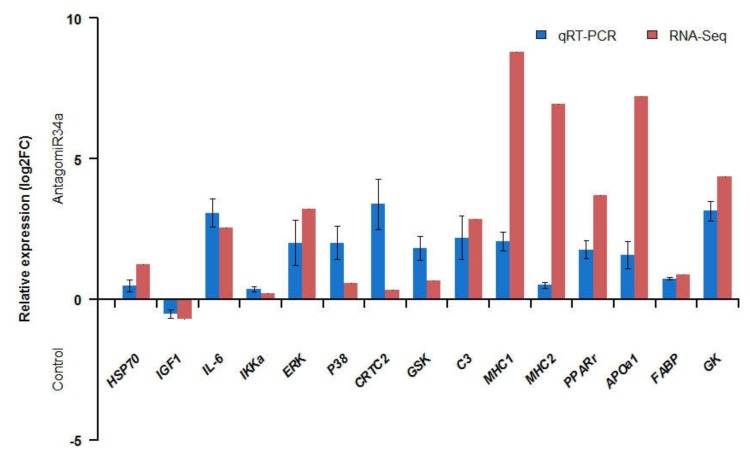
Gene expression patterns of RNA-Seq and qRT-PCR. *β-actin* was used as an internal control and used for the normalization of the expression level of each gene. Log-fold changes are expressed as the ratio of gene expression. Error bars represent standard error.

To validate the reliability of RNA-seq in this study, the expression level of 15 glucose metabolic-related genes were analyzed by qRT-PCR. As shown in Figure 5, the qRT-PCR results were in high accordance with the RNA-seq analysis. All genes were in consistent with the RNA-seq results. The results showed that the expression patterns of these genes were in consistent in two methods, which was helpful for confirming the validity of our analysis of glucose metabolism in *M. amblycephala*.

### 2.7. Gene ontology (GO) and Kyoto Encyclopedia of Genes and Genomes (KEGG) Pathways Enrichment Analysis of differentially expressed genes (DEGs)

**Figure 6:**
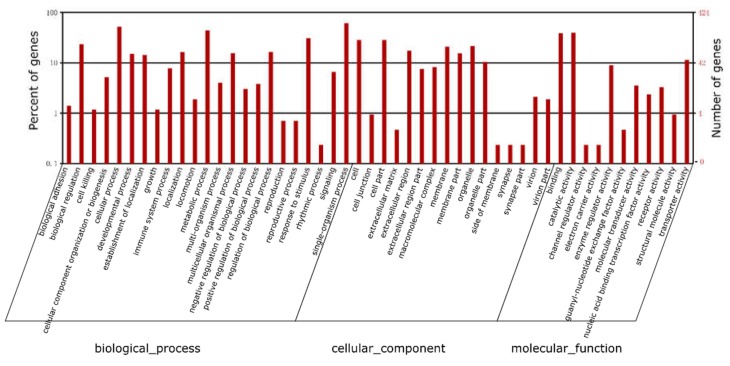
Gene ontology enrichments of all differentially expressed genes responded to miR-34a inhibitor treatment.

**Figure 7:**
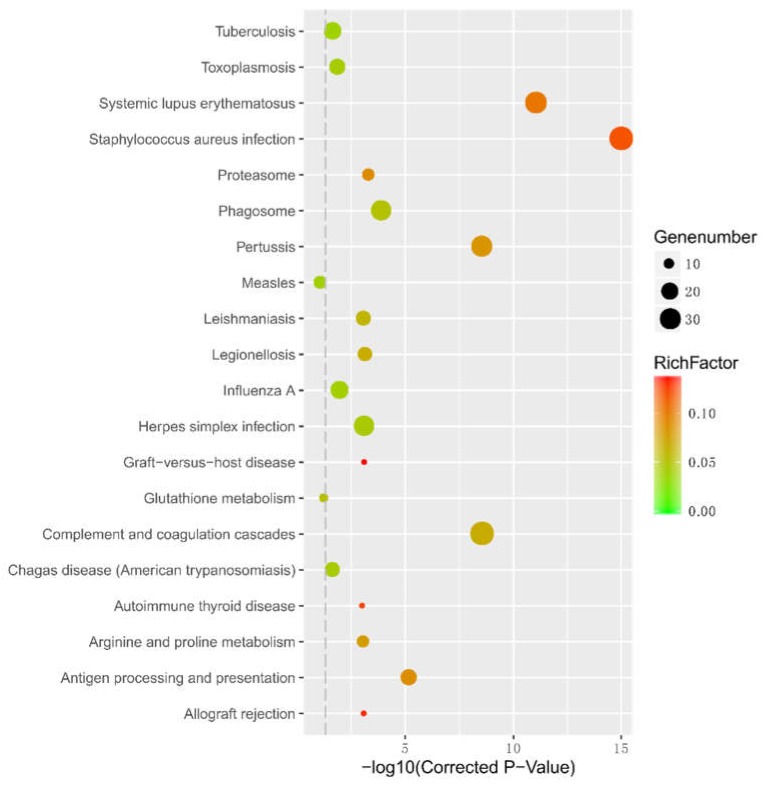
Glucose metabolic-related KEGG pathways for differentially expressed genes responded to miR-34a inhibitor treatment.

In order to obtain the potential function annotation of all DEGs, further enrichment analysis of the GO terms and KEGG pathways were performed (Figure 6 and Figure 7, and Table S4). Based on the GO functional annotation, we found DEGs were classified into decades of molecular functions, cellular components and biological processes (corrected *p* < 0.05, Figure 6 and Table S4), including somelipid, glucose, insulin metabolic-related terms (i.e., lipid metabolic process (GO:0006629), cholesterol metabolic process (GO:0008203), glucose metabolic process (GO:0006006), response to lipid (GO:0033993), carboxylic acid metabolic process (GO:0019752) and carboxylic acid transport (GO:0046942)). For example, some genes including downregulated *PEPCK*, *PKM* (pyruvate kinase PKM-like), and *INSIG1* were enriched into these GO terms.

KEGG pathway enrichment analysis showed that the most significant enriched signal pathway was JAK-STAT signaling pathway (ko04630) (Figure 7 and Table S4). It has been reported that the JAK-STAT pathway was responded to the metabolism in both type 1 diabetes (T1D) and T2D [29]. Obesity is a major risk factor for development of T2D, and lacking of leptin or the leptin receptor (*LepRb*/*ObRb*) will develop severe obesity and insulin resistance. When leptin binds to the β-isoform of the leptin receptor, *JAK2* is activated by autophosphorylation to phosphorylate tyrosine residues on the cytoplasmic tail of the receptor [30]. The enriched DEGs, such as *STAT1* (signal transducer and activator of transcription 1), were upregulated and the leptin was downregulated in antagomiR34a-12h samples, which was in coincidence with the former research [29,30]. In addition, we found some notable pathways, including type I diabetes mellitus (ko04940), type II diabetes mellitus (ko04930), PPAR signaling pathway (ko03320), fatty acid biosynthesis (ko00061), fat digestion and absorption (ko04975), p53 signaling pathway (ko04115), insulin signaling pathway (ko04910), PI3K-Akt signaling pathway (ko04151), glycolysis/gluconeogenesis (ko00010) and insulin secretion (ko04911), were associated with these DEGs. DEGS were found to be significantly enriched into glucose/insulin related pathways, in which upregulated *MHC1* and *CREBBP* and downregulated *MHCII* and *PIK3R1* serve as key regulatory proteins. For instance, *MHC1*, a T1D related genes [31], was found upregulated in the antagomiR34a-12h sample.

### 2.8. Change of Some Other Glucose Metabolism Related Genes after Inhibition of miR-34a

**Figure 8:**
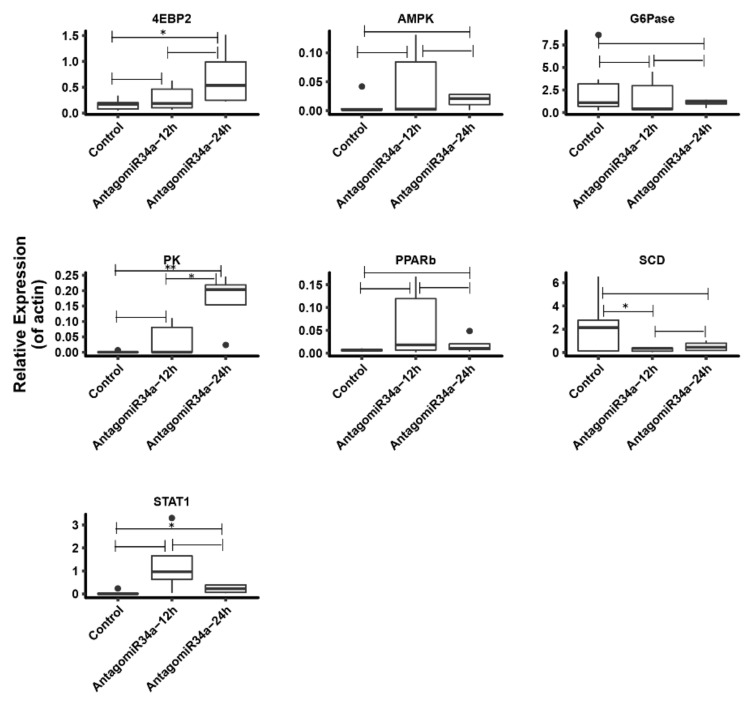
qRT-PCR analysis of some glucose metabolism-related genes involved in AMPK signaling pathway. * *p* < 0.05; ** *p* < 0.01. Data not included between the whiskers was plotted as an outlier with black dot.

The RNA-seq results showed that the mRNA levels of some gluconeogenic genes were not significantly differentially expressed, such as *PPARb* (Peroxisome proliferator-activated receptor b), *4EBP2* (Eukaryotic translation initiation factor 4E-binding protein 2), *AMPK* (AMP-activated protein kinase), *STAT1*, *PKM* (pyruvate kinase PKM-like isoform X1), *G6Pase* (Glucose6-phosphatase), and *SCD* (Stearoyl-CoA Desaturase). So we carried out additional qRT-PCR analysis to detect their expression levels in livers of *M. amblycephala* treated with miR-34a inhibitor or control. In addition, we planned to determine if these dysregulations were progressive with treatment time or not. Changes in the relative mRNA levels of these genes were shown in Figure 8. The expression of *PPARb*, *4EBP2*, *AMPK*, *STAT1* and *PKM* were increased by antagomiR34a treatments comparing with control, and the expression levels of *G6Pase* and *SCD* were downregulated in antagomiR34a-12h group in comparison with control. We also found that 3/7 tested genes were increased in liver samples at 24 h post antagomiR34a treatment comparing with those at 12 h. These might suggested that glucose related genes were progressively accumulated in livers by miR-34a inhibitor injection into *M. amblycephala.*

## 3. Discussion and Conclusions

*M. amblycephala* is a widely cultured freshwater fish in China, but the molecular mechanism of nutritional utilization of dietary carbohydrates is still unclear [32]. Previous studies had demonstrated that altered dietary and metabolic conditions were affected by gene regulation in the cell nucleus [24]. We had previously performed a next-generation sequencing study between normal starch diet and high starch diet treated fishes, and identified hundreds of DEMs that responded to HSD treatment in intestine, liver, and brain in *M. amblycephala*, respectively, suggesting that miRNAs might play crucial roles in glucose metabolism [9]. Among the DEMs, we found that the miR-34a was significantly upregulated in HSD group. miR-34a was a key regulator in the glucose metabolism [17,18,19,20,21], and miR-34a upregulated expression had been identified in T2D patients compared to health controls [21]. A plausible molecular mechanism for the role and function of miR-34a in metabolism was the loop of miR-34a, *SIRT1* and *p53* [33]. But how miR-34a affected the course of glucose intolerance responses in *M. amblycephala* was still unknown.

So we conducted an in vivo experiment with intraperitoneally injection of miR-34a inhibitor into *M. amblycephala*, and identified the DEGs related to miR-34a inhibition. The qRT-PCR analysis showed that miR-34a was significant inhibited at 12, 24 and 48 h post the antagomiR34a treatments, with dysregulation of 2212 DEGs. GO and KEGG pathway analysis showed that the DEGs were enriched in the signaling pathways, including PPAR pathway, insulin signaling pathway, JAK/STAT signal pathway, Type I diabetes mellitus and Type II diabetes mellitus, which were associated with glucose/lipid metabolic pathways and biosynthetic processes. All these results implied that the inhibition of miR-34a could regulate a series of genes, which might be crucial for glucose metabolism in *M. amblycephala*.

Previous studies have demonstrated that miR-34a could reduce the expression level of *SIRT1* and prevent the activation of *PGC-1α*, *PPARα*, *p53* and *FOXO1* to alter the expression of transcriptional progresses and supervise lipid/glucose, cholesterol and energy homeostasis [34,35,36,37,38,39,40,41]. But the expression levels of these genes were not significantly differentially expressed in our study. We found that one of the PPARs family members, *PPARb*, was upregulated in antagomiR34a treated samples by qRT-PCR (Figure 8). *PPARb* was a nuclear hormone receptor which governs a variety of biological processes and may be involved in the development of several diabetes and obesity diseases [42,43]. For example, Sanderson et al., proved that *PPARb*/*δ* deletion could downregulate the pathways associated with lipoprotetin metabolism and various pathways related to glucose utilization [44]. Consistent with these results, we found *PPARb* was upregulated in samples injected with miR-34a inhibitors, implying that the deletion of miR-34a could upregulate *PPARb* and the pathways associated with the glucose metabolism in *M. amblycephala*. These might provide a potential novel role of miR-34a in the regulation of glucose metabolism in *M. amblycephala*.

**Figure 9:**
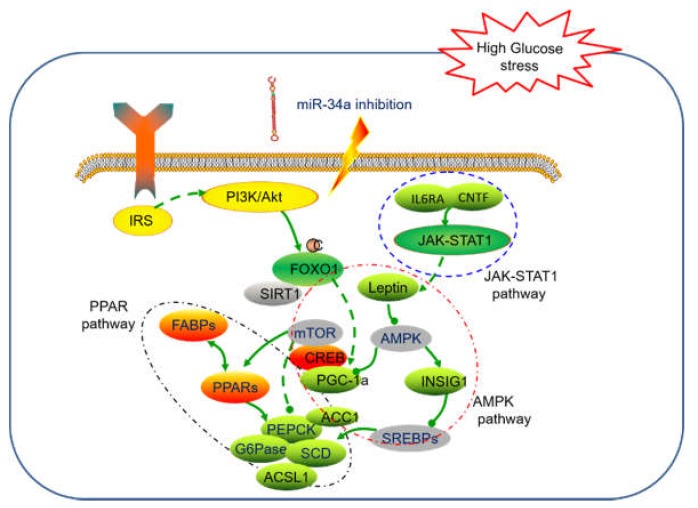
The potential interaction and molecular mechanism of putative genes involved in glucose metabolism process regulated by miR-34a inhibition. Green indicates downregulation, while and red and yellow note upregulation in our study. Gray notes no detection of dysregulation. The blue, red and grey circles include related genes in JAK-STAT1 pathway, AMPK pathway and PPAR pathway, respectively. The green line with an arrow and the green line with a ball stand for positive and negative effect between genes, and the green dot line with an arrow and the green dot line with a ball stand for supposed positive and negative effect between genes.

In addition to the genes involved in the putative regulatory loop of miR-34a and *SIRT1* (Figure 9) [45], other regulators responded to insulin and glucose homeostasis were found dysregulated in the analysis of RNA-seq data. For example, *NSIG1*, the negative regulator of SREBPs, was downregulated in antagomiR34a-12h sample. Previous study showed that the overexpression of *INSIG1* could significantly inhibit *SREBP-1c* expression and thereby blocking the expression of downstream genes related to insulin and lipid metabolic pathways (Figure 9) [46]. Consistent with this, the downregulated *INSIG1* was detected in antagomiR34a-12h sample, which might increase the expression of SREBPs and the downstream genes. We also found that *PLCB* (phosphatidylinositol phospholipase C, β), *GADD45A* (Growth arrest and DNA-damage-inducible protein GADD45 α), and *FABP* (fatty acid-binding protein) and *ACSL1* were downregulated after inhibiting miR-34a in *M. amblycephala* (Table S3). *ACSL1* is one of the long chain acyl-CoA synthetases in lipid metabolism and also been implicated in the cellular uptake of fatty acids. The overexpression of *ACSL1* showed increased acyl-CoA synthetase activity and fatty acid uptake [47]. In this study, the *ACSL1* was found downregulated in antagomiR34a-12h sample comparing with control. These results implied that the inhibition of miR-34a might play potential novel roles in glucose homeostasis in *M. amblycephala* by directly or indirectly regulating these key genes.

The DEGs in response to miR-34a inhibition in *M. amblycephala* livers was associated with JAK/STAT and PPAR signaling pathways. Some not significantly enriched pathways, including insulin-signaling pathway, Type I diabetes mellitus and Type II diabetes mellitus, were also found in the result of KEGG analysis. These pathways were related to DEGs including upregulated *PPP1C* (serine/threonine-protein phosphatase PP1 catalytic subunit) and *IL6RA* (interleukin 6 receptor α, downregulated) and downregulated *CNTF* (ciliary neurotrophic factor). Elevated *IL6* had been reported in diabetes mellitus type 2 [48]. We checked the expression level of *IL6* and found that *IL6* was upregulated in the antagomiR34a-treated sample (Figure 5). All these results suggested that inhibited miR-34a might regulate the glucose metabolism by altering pathways related to glucose utilization.

To avoid the incomplete results of RNA-seq from being less sequences samples, we carried out an additional qRT-PCR analysis to detect the expression profiles of glucose metabolism related genes. The results showed that the profiles of 7 selected key genes were all as expected and most of these genes were involved in the AMPK signaling pathway (Figure 8 and Figure 9). *AMPK* is an energy sensor that regulates cellular metabolism which could stimulate glucose uptake to produce energy when activated by a deficit in nutrient status [49]. Evidence has shown that gluconeogenesis in the liver is regulated by multiple enzymes such as *PEPCK* and *G6Pase* (Figure 9) [50], and the activation of *AMPK* could suppress the transcription of *G6Pase* in hepatoma cells [51]. Andreelli et al., suggested that there was glucose intolerant and fasting hyperglycemia in *AMPK* α2 liver-specific knockout mice, which presumably due to the increased *PEPCK* and *G6P*ase activity [52]. In our study, *AMPK* was found to be upregulated in the miR-34a inhibitor-injected samples and the expression level of G6Pase was decreased by qRT-PCR analysis (Figure 8). Another gene *PPARb* was involved in the glucose metabolism by activating a program that increased the coupling of glycolysis to glucose oxidation in muscle via the cooperation with AMPK signaling pathway to activate *Ldhb* gene transcription [53]. As expected, the relative expression level of *PPARb* was found to be increased in the miR-34a-inhibited group by qRT-PCR analysis, which revealed that the knock-down of miR-34a might upregulate *PPARb* expression, which then subsequently interacted with *AMPK* to regulate the glucose metabolism in the liver tissue in *M. amblycephala.* In addition, the expression level of 3/7 selected AMPK signaling pathway related genes showed a progressively accumulation along with handling time post injection of miR-34a inhibitor (Figure 8). These results implied that miR-34a could interact with AMPK signaling pathway to regulate the glucose embolism in *M. amblycephala.*

All the results in this study showed that the miR-34a was an important regulator in the glucose metabolism by activating or inactivating the downstream genes involved in the glycometabolism. The regulation of the expression level of miR-34a or its targets genes might provide a novel regulatory role in glucose metabolism in *M. amblycephala.*

## 4. Material and Methods

### 4.1. Ethics Statement

All experimental protocols, methods and feeding scheme were approved on October, 2017 by the Institutional Animal Care and Use Committee of Freshwater Fisheries Research Center, Chinese Academy of Fishery Sciences (Wuxi, China) in this study. All fishes were anesthetized in well-aerated water with 0.01% tricaine methanesulfonate (MS-222; Sigma, Saint Louis, MO, USA) and sacrificed according to the Guide for the Care and Use of Laboratory Animals of China.

### 4.2. Sample Collection

*M. amblycephala* were obtained from the Freshwater Fisheries Research Center (FFRC) in Wuxi, Jiangsu province, China. 120 healthy and young fishes (initial weight: 27.54 ± 0.16 g) were maintained in conical fiberglass tanks (volume: 300 L) in a flow-through water system (speed: 3 L/min) during the acclimation and experimental period. Water temperature was maintained at 26.0 ± 1.5 °C, pH = 6.8–7.0, dissolved oxygen ≥ 6 mg·L^−1^, ammonia nitrogen ≤ 0.2 mg·L^−1^, nitrite ≤ 0.02 mg·L^−1^. Aeration was supplied to each tank 24 h per day and natural photoperiod was followed. All fishes were fed with high starch diet (45% wheat starch, HSD). The formulation and proximate composition of the HSD were shown in Table 5.

After the preliminary breeding of 8 weeks, 60 fishes were randomly selected for further researches. Thirty fishes were intraperitoneally injected with miR-34a inhibitors (5′-ACAACCAGCUAAGACACUGCCA-3′, antagomiR-34a; 80 nmol/100 g body weight (RIOBIO, Guangzhou, China), and the other 30 fishes were intraperitoneally injected with miR-34a inhibitor negative control (5′-UGUUGGUCGAUUCUGUGACGGU-3′, NC; 80 nmol/100 g body weight; RIOBIO, Guangzhou, China) and were set as control group. Nine fishes injected with antagomiR-34a NC and antagomiR-34a at 12 h (antagomiR-34a-12h), 24 h (antagomiR-34a-24h) and 48 h (antagomiR-34a-48h) were collected, respectively, and anaesthetized using MS-222. The liver tissues were stripped immediately, stored at −80 °C and prepared for RNA isolation.

### 4.3. qRT-PCR Analysis for the miR-34a

Total RNA was extracted from *M. amblycephala* samples using TRizol (Invitrogen, Carlsbad, CA, USA) according to the manufacturer’s protocol. RNA purity was assessed using the NanoDrop-2000 (Thermo Fisher Scientific, BRIMS, Cambridge, MA, USA). RNA samples with A260:A280 ratios above 1.9 and A260:A230 ratios above 1.8 were used for reverse transcription with miRNA specific stem-loop primers (Table S5) and the PrimeScript RT Reagent kit (Takara Bio, Dalian, China). QRT-PCR analyses were performed according to methods described by Miao et al. [9]. The relative expression level of the miR-34a was calculated in terms of threshold cycle value (*C*_t_) and normalization to the expression of 5S rRNA using the equation 2^−ΔΔ*C*t^, where ∆*C*_t_ = *C*_t miRNA_ − *C*_t 5s_.

### 4.4. cDNA Library Construction and Sequencing

Total RNA was extracted as described above. Three samples (control-1, control-2 and antagomiR-34a-12h) with the RNA integrity Number (RIN) larger than 7.0 were selected for transcriptome sequencing. Next, three sequencing libraries were constructed by TruSeq^TM^ RNA Sample Preparation Kit according to the product instruction (Illumina, San Diego, CA, USA). Each library was sequenced using Illumina HiSeq 4000 for 2 × 150 bp pair-end (PE) sequencing.

### 4.5. De Novo Assembly of Reference Sequences

Fastqc (http://www.bioinformatics.babraham.ac.uk/projects/fastqc/) software was used to perform the quality control of all raw reads. In this filtering step, we excluded the poor quality reads, including adaptor reads or unknown base more than 10% and low-quality reads (reads having more than 50% bases with quality value ≤5). Then the clean reads were firstly de novo assembled into contigs by Trinity software (version trinityrnaseq-2.0.2) with default parameters [55]. Finally, the obtained non-redundancy assembled contigs were clustered using CD-Hit (version v4.6.4) at 90% similarity and the remaining contigs were used for downstream analysis [56]. The resulting contigs were considered as the *M. amblycephala* reference sequences.

### 4.6. Assembled Sequence Annotation and Classification

BLASTx(version 2.5.0) alignment with a cut-off *e*-value < 1 × 10^−10^ was performed to search against four public database, including NCBI Nr (Non-redundant protein) database [57], the UniProt-SwissProt (The Universal Protein Resource) database [58], the COG database [59], Pfam database [60] and the KEGG database [61]. The gene name and description of the best blast hit was assigned to each contig.

### 4.7. SSRs Identification

MIcroSAtellite (MISA) (http://pgrc.ipk-gatersleben.de/misa/misa.html) was used to detect the SSR motifs in all assembled contigs with the parameters of at least 6 repeats for di-nucleotide and 5 repeats for tri-, tetra-, penta- and hexa-nucleotide. The compound motifs containing more than one microsatellite sequences separated by 100 bases were also identified.

### 4.8. ORF Identification and Prediction

We used an in-house Perl scripts to obtain the ORF regions within the contigs which had hits against the Nr database. For the unannotated contigs, we used Transdecoder (http://transdecoder.sourceforge.net/) program to predict the putative ORFs regions. Full-length ORF region were defined as the ORF region of a contig can cover the entire length of the subject protein. As for the other case, the completeness percentage was calculated by the subject protein coverage.

### 4.9. Identification of DEGs

Bowtie (version 1.0.0) [62] and RSEM (version 1.2.21) [63] program were used to quantify the abundances for all genes and isoforms in two groups. Gene expression was normalized by FPKM (fragments per kilobase of exon per million reads mapped). Then, edgeR (version 3.10.5) [64] was used to identify the DEGs by pairwise comparisons in the control and antagomiR-34a-12h samples. The cut-off criteria for DEGs were |logFC| ≥ 1 and FDR ≤ 0.05 between two groups. GO term annotation was performed using the GO::TermFinder program (version 0.86) to unravel the statistically significant enrichment of DEGs with a correct *p*-value cutoff of 0.05 [65]. KEGG metabolic pathway analysis was conducted using the KOBAS program (version 2.0), taking the correct *p*-value ≤ 0.05 as a threshold to identify enrichment pathway [66].

### 4.10. QRT-PCR Analysis for DEGs

Total RNA was extracted as described before and the primers of all selected genes were designed (Table S1). qRT-PCR was performed on the ABI PRISM 7500 real-time PCR System (Applied Biosystems, USA). The amplifications were performed in a total volume of 10 μL and included 5 μL of 2X SYBR Green MasterMix reagent (Thermo Fisher Scientific, Rockford, IL, USA), 1 μL of cDNA and 0.2 μL of each primer (10 μM). The thermal cycling profile consisted of an initial denaturation at 95 °C for 5 min followed by 40 cycles of denaturation at 95 °C for 15 s and annealing/extension at 60 °C for 45 s. An additional temperature-ramping step from 95 to 65 °C was performed to enervate the melting curve. All samples were examined in triplicates of two biology replicates and accompanied with negative controls. The β-actin was used as the internal control and the relative expression was calculated by the 2^−ΔΔ*C*t^ method [39].

### 4.11. Statistical Analysis

The statistical analysis of miR-34a in fish livers by qRT-PCR was analyzed using student’s *t*-test in Graphad prism 6.0 (Graphpad Software, San Diego, CA, USA). *p* < 0.05 was set as the criteria for statistically significant.

## Figures and Tables

**Table 1:** Summary of the sequencing data from *M. amblycephala*.

Sample	Raw Reads	Clean Reads	Clean Q20 (%)	Clean Q30 (%)	Clean GC (%)
AntagomiR34a-12h	40,929,414	40,359,036	97.33	92.81	46.96
Control-1	51,674,516	50,991,682	97.39	92.97	46.35
Control-2	52,931,508	52,218,818	97.47	93.14	47.20

Note: AntagomiR34a-12h: miR-34a knocked down for 12 h sample, control-1 and control-2 were high-starch samples.

**Table 2:** Overall assembly statistics for the transcriptome of *M. amblycephala.*

Item	Value
Total number of sequences	222,411
Total number of genes	197,425
Total length of sequences (bp)	151,079,306
Maximum sequence length (bp)	16,839
Minimum sequence length (bp)	200
N50	1041

**Table 3:** Statistics of function annotation.

Annotated Database	Annotated Number	Transcript Ratio (%)
All_Assembly_Contig	222,411	100
Nr	82,223	36.97
SwissProt	57,581	25.89
KEGG	71,045	31.94
KOG	45,994	20.68
Pfam	27,677	12.44
GO	25,433	11.44
All_anno_contig	83,587	37.58

**Table 4:** Distribution of simple sequence repeat (SSR)s based on the number of repeat units.

Repeat Numbers	SSR Type						Total
	Mono-	Di-	Tri-	Tetra-	Penta-	Hexa-	
5	0	0	3436	996	154	21	4607
6	0	4847	1545	710	38	11	7151
7	0	2673	948	75	18	1	3715
8	0	1674	873	93	24	1	2665
9	0	1266	134	60	35	0	1495
10	11,710	1138	198	60	22	1	13,129
11	7843	2036	129	38	24	0	10,070
12	5217	1165	84	27	27	0	6520
≥13	37,415	5640	172	190	64	1	43,482
Total	62,185	20,439	7519	2249	406	36	92,834
%	66.99%	22.02%	8.10%	2.42%	0.44%	0.04%	

**Table 5:** Formulation and proximate composition of the HSD (%).

Ingredient	Quantity (g)	Proximate Composition	(% of Dry Matter)
Casein ^1^	20	Crude protein	33.32
Gelatin	5	Crude lipid	8.58
Fish meal ^2^	16	Digestible carbohydrate	45.25
Wheat starch ^3^	45	Energy (kJ·g^−1^)	18.55
Soybean oil ^4^	7	Calcium	1.06
Vitamin additive ^5^	1	Total phosphorus	1.32
Mineral additives ^5^	1		
Carboxymethycellulose	4		
Monocalcium phosphate ^5^	1		

Note: ^1^ Provided by Provided by Feeer Co., Ltd. (Shanghai, China), protein content 88.7%; ^2^ Provided by Tongwei Co., Ltd. (Wuxi, China). Protein content 61.2%; ^3^ Provided by Jinglingta Co., Ltd. (Wuxi, China); ^4^ Fat acid content of 0.096% C14:0, 11.479% C16:0, 0.105% C16:1, 0.095% C17:0, 4.264% C18:0, 20.132% C18:1, 55.706% C18:2, 7.247% C18:3n3, 0.319% C20:0, 0.246% C20:1 and 0.313% C22:0; ^5^ Provided by Wuxi Hanove Animal Health Products Co., Ltd. (Wuxi, China). Vitamins premix and mineral premix was as referred to Ren et al. [54].

## Supplementary Materials

Supplementary materials can be found at www.mdpi.com/1422-0067/19/8/2417/s1.

## Abbreviations

FAO	Food and Agriculture Organization
DEM	Differentially expressed miRNA
T2D	Type 2 diabetes
NAFLD	Non-alcoholic fatty liver disease
ACSL1	Acyl-CoA synthetase long-chain family member 1
FOXO1	Forkhead box protein O1
PPARs	Peroxisome proliferator-activated receptors
PGC-1α	Peroxisome proliferator-activated receptor γ coactivator 1-α
COG	Cluster of Orthologous Groups of protein
MISA	MIcroSAtellite
SSR	Simple sequence repeat
KEGG	Kyoto Encyclopedia of Genes and Genomes
ORF	Open reading frame
FPKM	Fragments per kilobase of exon per million reads mapped
FDR	False Discovery Rate
HSD	High starch diet
