# Supplementary figures and images for: In Vivo Analysis of miR-34a Regulated Glucose Metabolism Related Genes in Megalobrama amblycephala
Source: Int J Mol Sci. 2018 Aug 16;19(8):2417. doi: 10.3390/ijms19082417 (PMC6121310; doi:10.3390/ijms19082417)

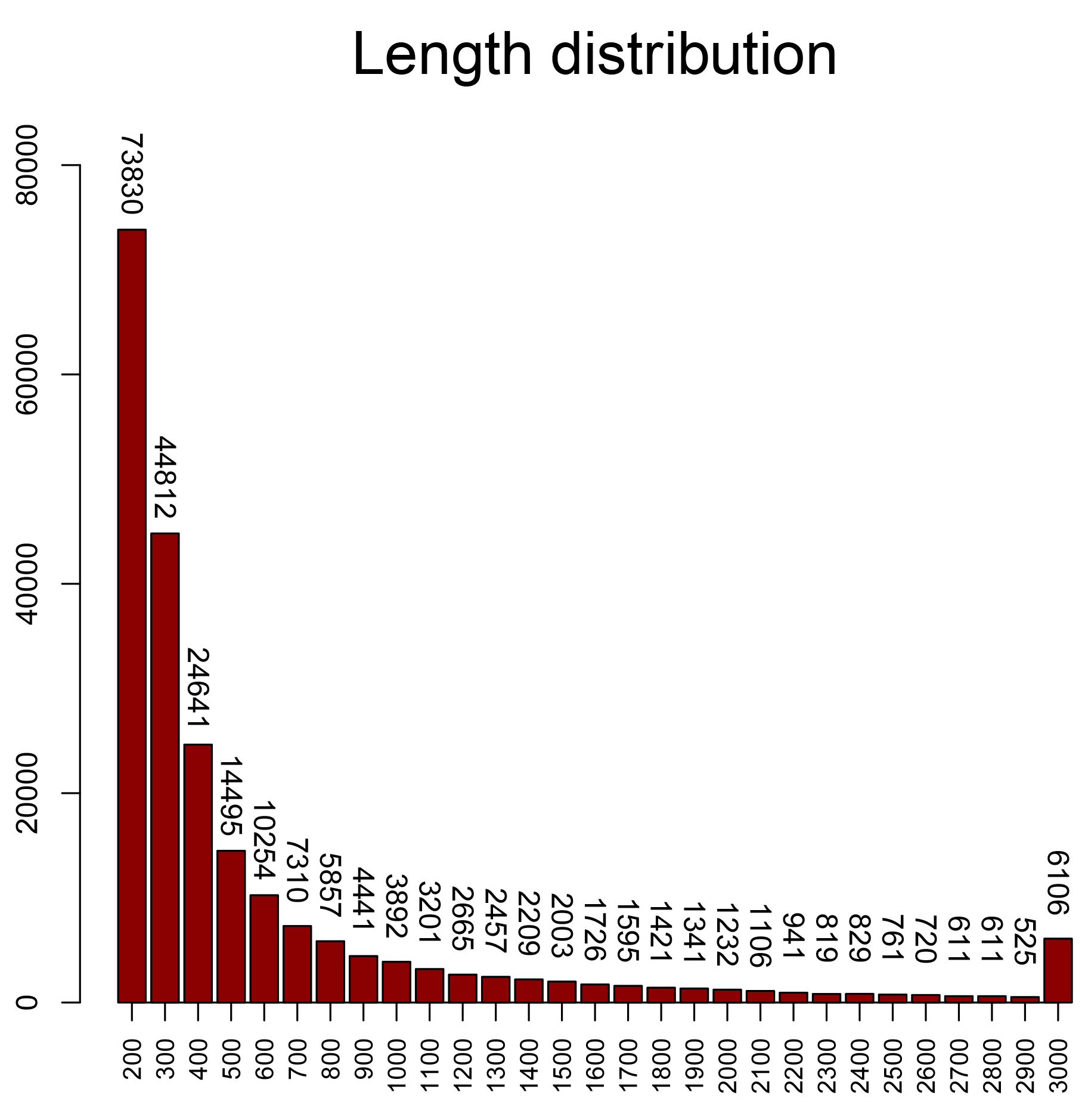

Supplement: Supplementary file 1 [file ijms-19-02417-s001.zip › supplementary file/Supplementary Figure S1.tif]

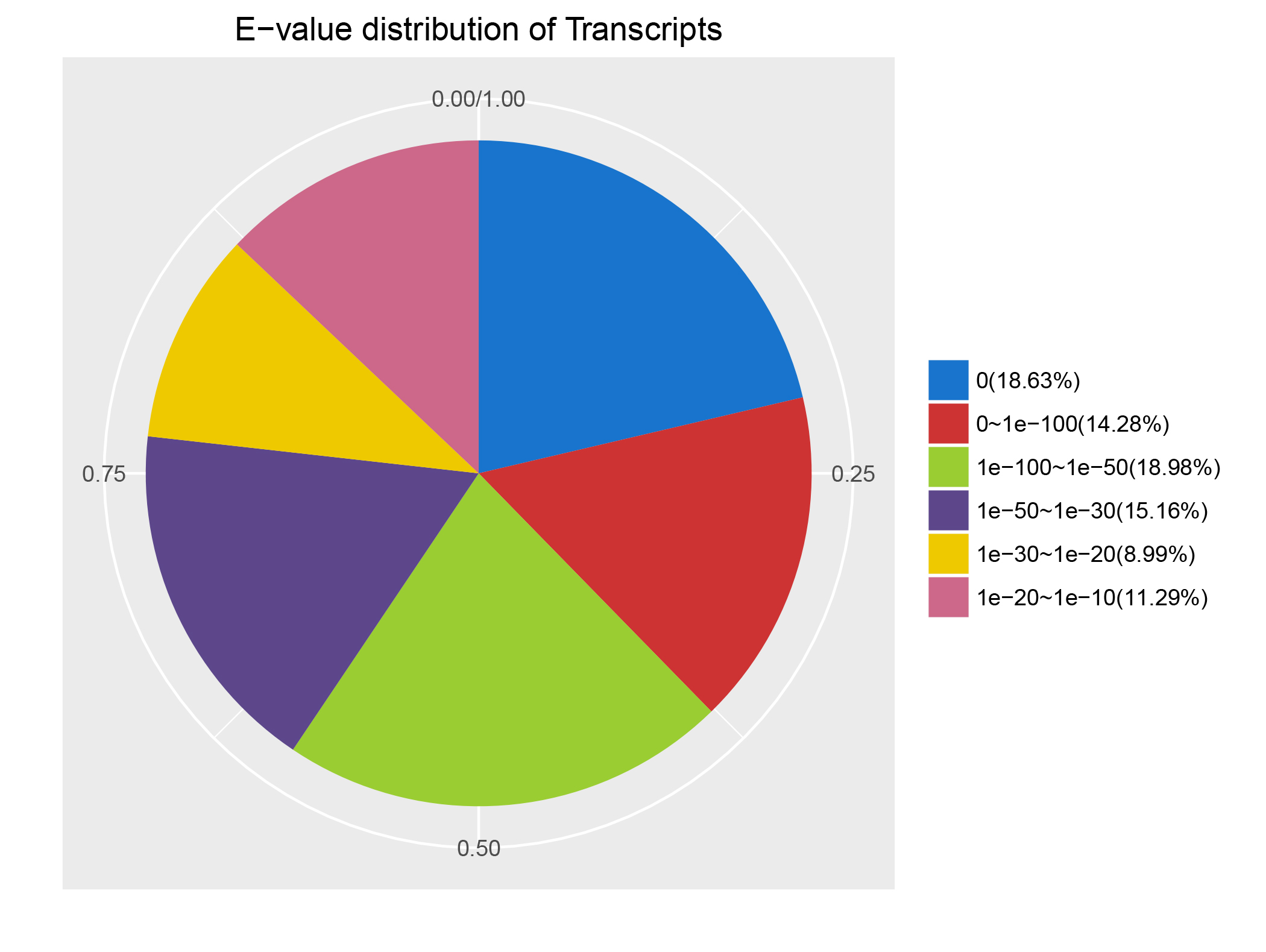

Supplement: Supplementary file 1 [file ijms-19-02417-s001.zip › supplementary file/Supplementary Figure S2.jpg]

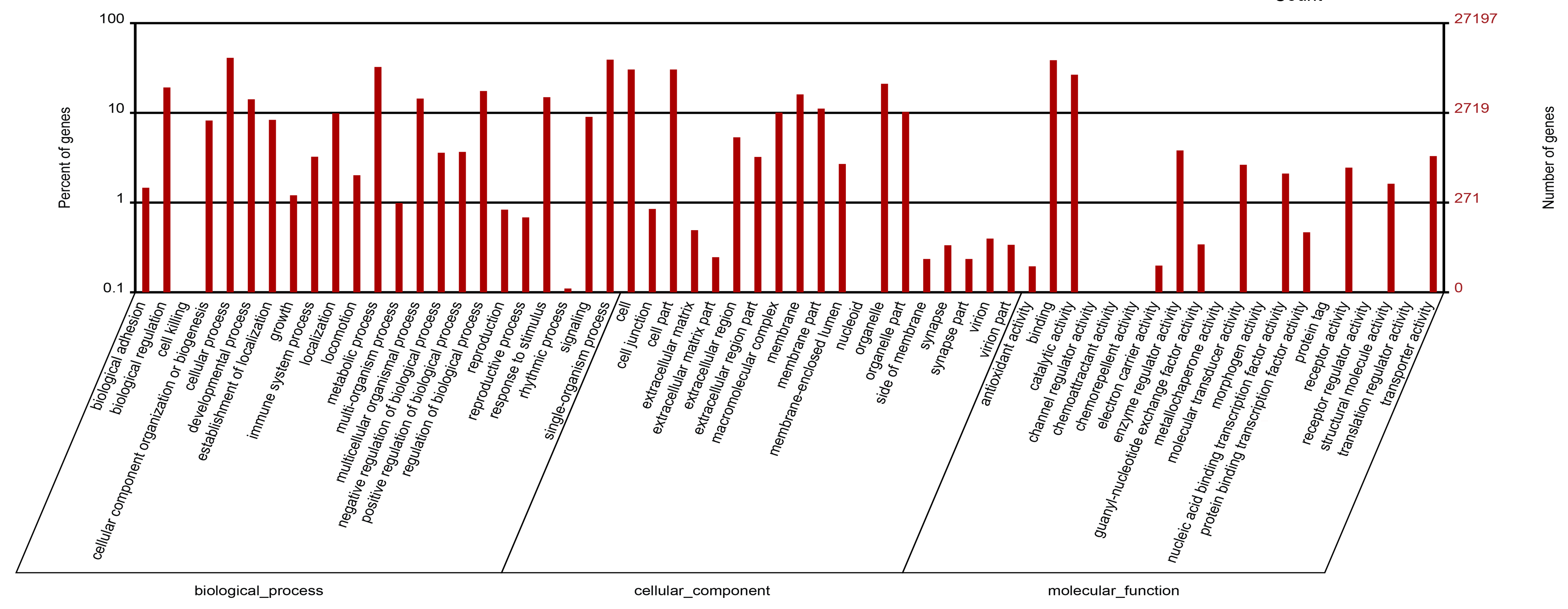

Supplement: Supplementary file 1 [file ijms-19-02417-s001.zip › supplementary file/Supplementary Figure S3.tif]

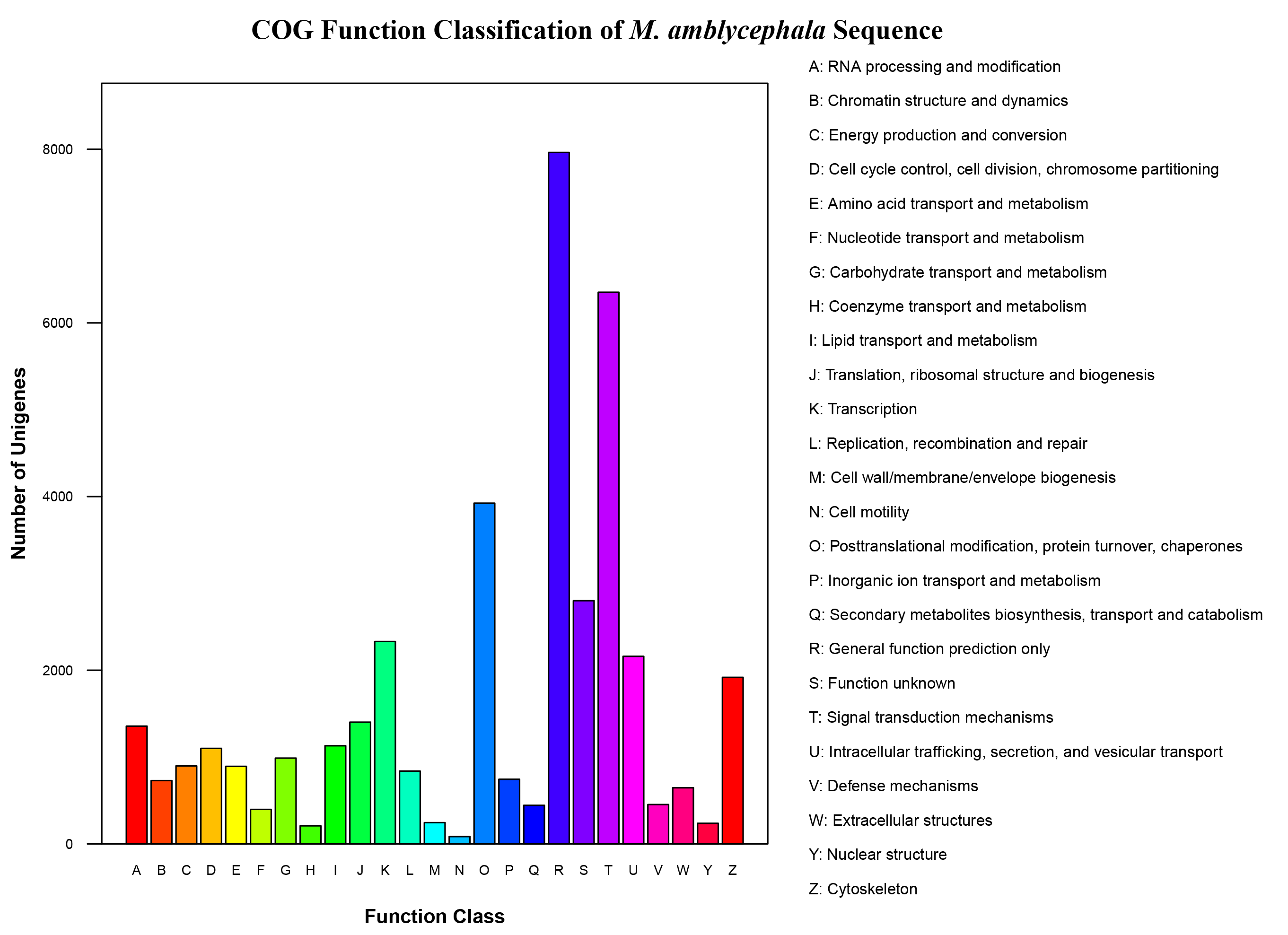

Supplement: Supplementary file 1 [file ijms-19-02417-s001.zip › supplementary file/Supplementary Figure S4.tif]

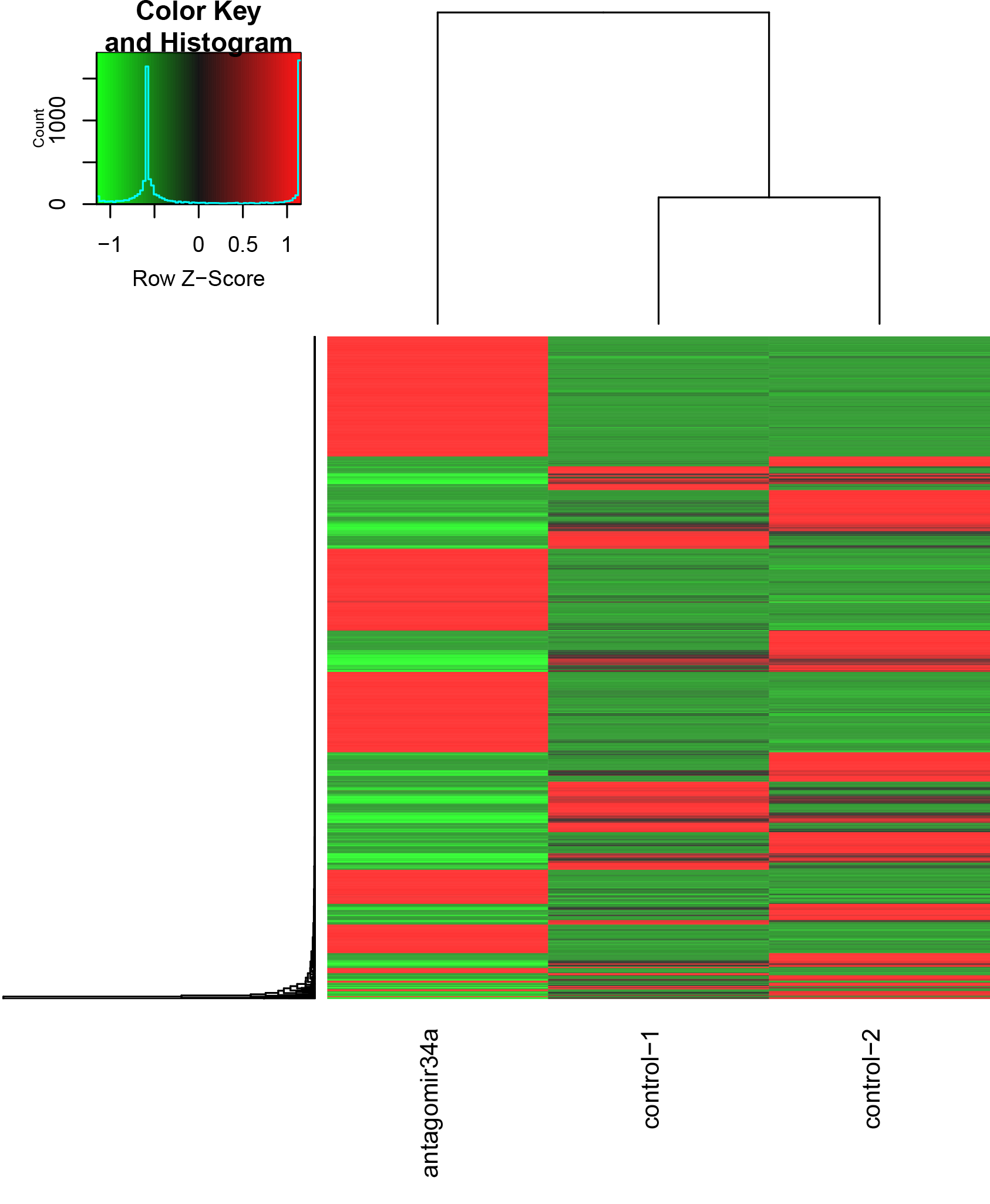

Supplement: Supplementary file 1 [file ijms-19-02417-s001.zip › supplementary file/Supplementary Figure S5.tif]
